# Omalizumab as an Effective Therapy for Testosterone-induced Angioedema in a Transgender Person: A Case Report

**DOI:** 10.2174/0118715303399441250701073138

**Published:** 2025-07-10

**Authors:** Lorenzo Marinelli, Giovanna Motta, Lorenzo Castella, Susanna Voltolini, Antonino Romano, Angelica Del Giudice

**Affiliations:** 1 Division of Endocrinology, Diabetes and Metabolism, Department of Medical Sciences, University of Turin, Turin, Italy;; 2 Allergy Outpatient Clinic, Genoa, Italy;; 3 Oasi Research Institute-IRCCS, Troina, Italy;; 4 BIOS S.p.A. Società Benefit, Rome, Italy;; 5 Department of Medicine, SS of Allergology, Martini Hospital, Turin, Italy

**Keywords:** Omalizumab, testosterone, angioedema, case report, drug allergy, transgender

## Abstract

**Background:**

Gender affirming hormone therapy is a cornerstone for transgender and gender diverse individuals. We report the first case in the literature of systemic allergic reactions to testosterone therapy and how we succeeded in managing them.

**Case Presentation:**

A 19-year-old transgender person assigned female at birth started testosterone therapy to achieve full masculinization. For 14 months, testosterone was administered *via* transdermal and intramuscular preparations and was well tolerated. However, the individual later developed several angioedematous reactions at different times, despite the use of various testosterone formulations. Allergy evaluations, such as blood tests, SPT, IDT, and drug challenges for both testosterone and excipients, were conducted; the drug challenge for testosterone was positive, confirming it as the trigger of angioedema. The use of omalizumab 150 mg subcutaneously every two weeks allowed the patient to resume testosterone therapy without further systemic reactions. This helped to reduce the patient’s perceived stress and supported his psycho-physical well-being.

**Conclusion:**

Omalizumab was able to successfully manage the systemic allergic reactions to testosterone therapy, and this report could be useful for other clinicians facing similar clinical situations.

## 1 INTRODUCTION

Transgender and Gender Diverse (TGD) people experience a marked and persistent incongruence between their perceived gender and the assigned gender at birth. To align their body characteristics with their gender identity, TGD individuals may ask for Gender-Affirming Hormone Therapy (GAHT) and gender-affirming surgeries [1]. For TGD people who are assigned female at birth (t-AFAB), GAHT is focused on inducing body virilization and defeminization using testosterone therapy. Testosterone can be delivered *via* short- or long-acting injections, transdermal gels or patches; however, testosterone therapies can have side effects, also from an allergic perspective [2].

Regarding the use of Testosterone Transdermal Delivery (TTD) systems, irritant contact dermatitis, sometimes severe (*e.g.*, with ulcers and blisters), is the most common type of adverse reaction [3, 4]. These systems are also responsible for cases of allergic contact dermatitis, in which T-cell-mediated hypersensitivity to testosterone has been diagnosed based on positive responses to patch tests [5, 6]. Local reactions to intramuscular testosterone injections have also been described [7]. With regard to systemic reactions, two anaphylactic events induced by the excipients of a long-acting intramuscular formulation of testosterone undecanoate (Reandron^®^, 1000 mg/4 ml) have been reported [8, 9]. In particular, the patients showed no reactivity to testosterone during allergy assessment, whereas Skin Prick Tests (SPTs) with benzyl benzoate and basophil activation with castor oil yielded positive responses, respectively.

The case we describe represents the first report of testosterone-induced angioedematous reactions and our approach to managing this occurrence successfully. This helped the patient continue his hormonal therapy to sustain his physical and mental well-being.

## 2 CASE REPORT

A 19-year-old t-AFAB individual referred to the local Gender Team in Turin, CIDIGEM (Interdepartmental Gender Dysphoria Center Molinette), to initiate GAHT in order to achieve full de-feminization and masculinization. After ruling out potential contraindications, a daily therapy with a transdermal formulation of 2% testosterone (Tostrex^®^) was started. This therapy was titrated up to a dose of 50 mg/day. After 6 months, following the person’s request, Tostrex^®^ was replaced with a long-acting intramuscular formulation of testosterone undecanoate (Nebido^®^, 1000 mg/4 ml) administered every 12 weeks, which allowed to achieve optimal serum testosterone levels (*i.e.*, 609 ± 123 ng/dl on average). During this period, the individual experienced several changes in his physical appearance, including hair growth, an increase in muscle mass, deepening of the voice, oily skin, cessation of the menses and clitoral enlargement. The first three injections were well tolerated. However, after the fourth administration, the patient suddenly began to present edema of the lips and eyelids at different time points. These manifestations subsided within a few hours following the oral administration of betamethasone (1.5 mg) and ebastine (10 mg). Since testosterone was not initially suspected as being responsible for the reaction, 12 weeks later, another dose of testosterone undecanoate was injected; about four hours after the administration, he experienced generalized pruritus and bilateral eyelid edema. Consequently, after the duration of action of Nebido^®^ had expired, the patient was re-administered Tostrex^®^ (40 mg/day), which was previously well-tolerated. After four days, the patient developed lip edema, which was responsive to corticosteroid and antihistamine therapy. Therefore, a different transdermal formulation that was not previously used was started, with a testosterone concentration of 20 mg/g (Testavan^®^). However, even with this product, edema of the eyelids and the feet was observed approximately 20 hours after the first application. Testosterone therapy was subsequently discontinued. His clinical history was revised, and no clinical conditions, no previous surgical interventions and the use of any other drugs were reported. In his family history, no one suffered from allergic conditions. Blood tests and allergy evaluations were performed to clarify the clinical case further and rule out possible causes of angioedema (Table **1**). SPTs were conducted using testosterone undecanoate (Nebido^®^) and a short-acting preparation not previously administered, containing a total of 250 mg of a mixture of four testosterone esters in 1 mL (Sustanon^®^: testosterone propionate 30 mg, phenylpropionate 60 mg, isocaproate 60 mg, and decanoate 100 mg). Both SPTs yielded negative results. Therefore, intradermal tests (IDTs) were performed by injecting 0.02 mL of Sustanon^®^ and Nebido^®^ diluted 1:1000, 1:100, and 1:10 in saline solution, all of which yielded negative results. SPTs conducted with the excipients of Nebido^®^ (*i.e.*, benzyl benzoate and castor oil) and Sustanon^®^ (*i.e.*, peanut oil and benzyl alcohol) were also negative. Both SPTs and IDTs with histamine (at 10 mg/mL and 1 mg/mL, respectively) produced wheals with maximum diameters of 5 mm and 8 mm, respectively, each surrounded by erythema.

Considering the growing amount of stress perceived by the lack of testosterone that increased gender dysphoria, an intramuscular Drug Challenge (DC) was performed to confirm or exclude testosterone hypersensitivity. On the first day, 1 ml of saline solution was injected intramuscularly, and no reaction occurred. On the following day, 0.1 ml of Sustanon^®^ was injected and tolerated, while on the third day, approximately 30 minutes after the 0.2 ml dose, the patient experienced bilateral eyelid edema, which regressed a few hours after the oral administration of betamethasone 1.5 mg and 10 mg of ebastine.

In light of the efficacy of omalizumab in the treatment of Chronic Spontaneous Urticaria (CSU) and its usefulness in drug desensitization procedures [10], therapy with omalizumab (Xolair^®^, 150 mg subcutaneously every two weeks) was undertaken. Before initiating the therapy as an off-label use, the patient was thoroughly informed about the potential benefits and risks associated with the drug, taking into account the available literature on drug desensitization. A written informed consent was obtained. Along with the fourth dose of omalizumab, Sustanon^®^ 250 mg intramuscularly every four weeks was restarted; furthermore, a daily tablet of ebastine 10 mg was added. Omalizumab 150 mg subcutaneously every two weeks was maintained. For six months, the patient did not report any further episodes of angioedema, regained progressively more energy, muscle strength, body virilization, and secondary amenorrhea. The drug was well tolerated, and no adverse reactions were observed. Starting from the seventh month, Sustanon^®^ was administered at a full-dose regimen every three weeks, and ebastine was lowered, being taken only the day before the testosterone injection.

**Fig. (1):**
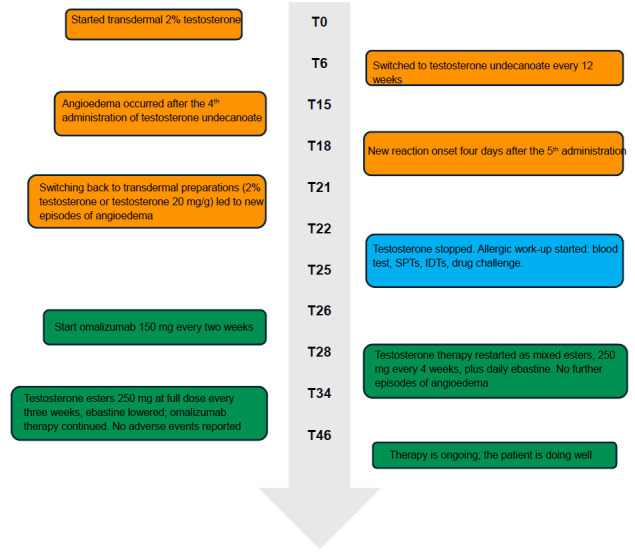
Timeline (in months) of the reported case involving a transgender individual assigned female at birth who developed angioedema after initiating testosterone therapy. **Abbreviations:** SPT: Skin prick test, IDT: Intradermal test

After a year, the patient is still on combined therapy with omalizumab with clinical stability. (Fig. **1**) presents a chronological graphical summary of the clinical case.

## 3 DISCUSSION

Among t-AFAB individuals, testosterone therapy plays a crucial role in supporting gender expression and improving body satisfaction. This enhances overall well-being, reduces perceived distress [1], and promotes a better overall quality of life—including sexual health— which are recognized by the World Health Organization as key components of general health [11]. Clinical history and positive DC indicated that testosterone was responsible for our patient's angioedematous reactions. To our knowledge, angioedema associated with testosterone therapy has not been reported yet. It was not possible to fully ascertain the pathogenic mechanism of these reactions. Negative testosterone skin tests would argue against the role for testosterone-specific IgE, although IgE- mediated hypersensitivity to testosterone metabolites cannot be completely excluded. Omalizumab, a humanized monoclonal antibody, primarily targets free serum IgE, preventing the interaction with the high-affinity IgE receptor (FcεRI) on mast cells and basophils. This results in subsequent downregulation of the FcεRI on such cells. Potential mechanisms of omalizumab in CSU may include reduction in the capability of mast cells to release mediators, as well as reduction in the intrinsically “abnormal” IgE activity and in coagulation abnormalities associated with disease activity [12]. Omalizumab has also been used successfully to enable desensitization in patients with IgE-mediated hypersensitivity to drugs [10], mainly platinum salts [13], or within the context of aspirin-exacerbated respiratory disease [14]. In CSU, omalizumab is used at a fixed dose of 300 mg every four weeks [12]. In some desensitization procedures, it was used at a fixed dose of 150 mg or 300 mg every second week, while in other cases the omalizumab dose was related to total IgE levels and body weight [10, 13]. Following the aforementioned literature, we decided to initiate omalizumab at a dose of 150 mg every two weeks. The therapy was well tolerated and successfully prevented the angioedematous flare-ups associated with testosterone treatment. To date, we have not discontinued the administration of omalizumab, as in patients with IgE-mediated drug hypersensitivity, it is typically maintained throughout both the desensitization procedure and the subsequent course of treatment with the relevant drugs [10].

Although the main limitation of this article is its nature as a case report, its value lies in being the first to describe testosterone-induced angioedema and a successful management strategy. Furthermore, these events affected a TGD individual for whom testosterone therapy was critical to sustaining psychological and physical well-being.

## CONCLUSION

This is the first reported case of angioedematous reactions to testosterone, regardless of the route of administration. Furthermore, this adverse reaction specifically occurred in a TGD person, and omalizumab allowed the continuation of GAHT with testosterone to guarantee the patient's psychophysical well-being. A future challenge will be to assess the effects of withdrawing omalizumab to determine whether testosterone therapy continues to trigger allergic manifestations.

## Figures and Tables

**Table 1:** Initial laboratory assessment.

-	**Reported Values**	**Normal Range Values**
Complete blood count-Eosinophils (cells/mm^3^)	100	30-500
CRP (mg/dl)	0.17	< 0.5
IgE (IU/ml)	30.17	< 90
IgM (mg/dl)	89	40-230
Tryptase (µg/L)	4.2	< 11.4
C3 complement (mg/dl)	90	90-180
C4 complement (mg/dl)	18	4-40
C1 inhibitor (%)	97	90-130
Serum protein electrophoresis	No monoclonal band	-

**Abbreviations:** CRP: C-Reactive Protein, IgE: Immunoglobulin E, IgM: Immunoglobulin M.

## Data Availability

All data generated or analyzed during this study are included in this published article.

## STANDARDS OF REPORTING

CARE guidelines and methodology were followed to conduct the study.

## References

[1] Coleman E., Radix A.E., Bouman W.P., Brown G.R., de Vries A.L.C., Deutsch M.B., Ettner R., Fraser L., Goodman M., Green J., Hancock A.B., Johnson T.W., Karasic D.H., Knudson G.A., Leibowitz S.F., Meyer-Bahlburg H.F.L., Monstrey S.J., Motmans J., Nahata L., Nieder T.O., Reisner S.L., Richards C., Schechter L.S., Tangpricha V., Tishelman A.C., Van Trotsenburg M.A.A., Winter S., Ducheny K., Adams N.J., Adrián T.M., Allen L.R., Azul D., Bagga H., Başar K., Bathory D.S., Belinky J.J., Berg D.R., Berli J.U., Bluebond-Langner R.O., Bouman M.B., Bowers M.L., Brassard P.J., Byrne J., Capitán L., Cargill C.J., Carswell J.M., Chang S.C., Chelvakumar G., Corneil T., Dalke K.B., De Cuypere G., de Vries E., Den Heijer M., Devor A.H., Dhejne C., D’Marco A., Edmiston E.K., Edwards-Leeper L., Ehrbar R., Ehrensaft D., Eisfeld J., Elaut E., Erickson-Schroth L., Feldman J.L., Fisher A.D., Garcia M.M., Gijs L., Green S.E., Hall B.P., Hardy T.L.D., Irwig M.S., Jacobs L.A., Janssen A.C., Johnson K., Klink D.T., Kreukels B.P.C., Kuper L.E., Kvach E.J., Malouf M.A., Massey R., Mazur T., McLachlan C., Morrison S.D., Mosser S.W., Neira P.M., Nygren U., Oates J.M., Obedin-Maliver J., Pagkalos G., Patton J., Phanuphak N., Rachlin K., Reed T., Rider G.N., Ristori J., Robbins-Cherry S., Roberts S.A., Rodriguez-Wallberg K.A., Rosenthal S.M., Sabir K., Safer J.D., Scheim A.I., Seal L.J., Sehoole T.J., Spencer K., St Amand C., Steensma T.D., Strang J.F., Taylor G.B., Tilleman K., T’Sjoen G.G., Vala L.N., Van Mello N.M., Veale J.F., Vencill J.A., Vincent B., Wesp L.M., West M.A., Arcelus J. (2022). Standards of care for the health of transgender and gender diverse people, version 8.. Int. J. Transgender Health.

[2] Hembree W.C., Cohen-Kettenis P.T., Gooren L., Hannema S.E., Meyer W.J., Murad M.H., Rosenthal S.M., Safer J.D., Tangpricha V., T’Sjoen G.G. (2017). Endocrine treatment of gender-dysphoric/gender-incongruent persons: An endocrine society clinical practice guideline.. J. Clin. Endocrinol. Metab..

[3] Wilson D.E., Kaidbey K., Boike S.C., Jorkasky D.K. (1998). Use of topical corticosteroid pretreatment to reduce the incidence and severity of skin reactions associated with testosterone transdermal therapy.. Clin. Ther..

[4] Lawrentschuk N., Fleshner N. (2009). Severe irritant contact dermatitis causing skin ulceration secondary to a testosterone patch.. ScientificWorldJournal.

[5] Buckley D.A., Wilkinson S.M., Higgins E.M. (1998). Contact allergy to a testosterone patch.. Contact Dermat..

[6] Shouls J., Shum K.W., Gadour M., Gawkrodger D.J. (2001). Contact allergy to testosterone in an androgen patch: Control of symptoms by pre-application of topical corticosteroid.. Contact Dermatitis.

[7] Spratt D.I., Stewart I.I., Savage C., Craig W., Spack N.P., Chandler D.W., Spratt L.V., Eimicke T., Olshan J.S. (2017). Subcutaneous injection of testosterone is an effective and preferred alternative to intramuscular injection: Demonstration in female-to-male transgender patients.. J. Clin. Endocrinol. Metab..

[8] Ong G.S.Y., Somerville C.P., Jones T.W., Walsh J.P. (2012). Anaphylaxis triggered by benzyl benzoate in a preparation of depot testosterone undecanoate.. Case Rep. Med..

[9] Santos-Vicente F., Latasa-Eceizabarrena M., Estrada-Rodríguez J.L., Pérez B., Sanz-Larruga M.L. (2021). The basophil activation test as an aid in the diagnosis and treatment of anaphylaxis associated with a testosterone preparation.. Rev. Alerg. Mex..

[10] Fernandez J., Ruano-Zaragoza M., Blanca-Lopez N. (2020). Omalizumab and other biologics in drug desensitization.. Curr. Opin. Allergy Clin. Immunol..

[11] Marinelli L., Cagnina S., Bichiri A., Magistri D., Crespi C., Motta G. (2024). Sexual function of transgender assigned female at birth seeking gender affirming care: A narrative review.. Int. J. Impot. Res..

[12] Agache I., Rocha C., Pereira A., Song Y., Alonso-Coello P., Solà I., Beltran J., Posso M., Akdis C.A., Akdis M., Brockow K., Chivato T., del Giacco S., Eiwegger T., Eyerich K., Giménez-Arnau A., Gutermuth J., Guttman-Yassky E., Maurer M., Ogg G., Ong P., O’Mahony L., Schwarze J., Werfel T., Canelo-Aybar C., Palomares O., Jutel M. (2021). Efficacy and safety of treatment with omalizumab for chronic spontaneous urticaria: A systematic review for the EAACI Biologicals Guidelines.. Allergy.

[13] Bumbacea R.S., Ali S., Corcea S.L., Spiru L., Nitipir C., Strambu V., Bumbacea D. (2021). Omalizumab for successful chemotherapy desensitisation: What we know so far.. Clin. Transl. Allergy.

[14] Lang D.M., Aronica M.A., Maierson E.S., Wang X.F., Vasas D.C., Hazen S.L. (2018). Omalizumab can inhibit respiratory reaction during aspirin desensitization.. Ann. Allergy Asthma Immunol..

